# Chimeric maternal cells as T lymphocyte targets in pediatric SLE

**DOI:** 10.1186/1546-0096-10-S1-A4

**Published:** 2012-07-13

**Authors:** Anne M Stevens, Matthew Crabtree, Gretchen Henstorf, J Lee Nelson

**Affiliations:** 1 Fred Hutchinson Cancer Research Center, Seattle, WA, USA; 2 Seattle Children's Research Institute, Seattle, WA, USA; 3 University of Washington, Seattle, WA, USA

## Purpose

Maternal cells routinely pass into the fetus, with increased frequency during complicated pregnancies. Engraftment of maternal cells into fetal organs such as heart and kidney may persist for decades after birth. Thus, SLE target organs normally harbor maternal cells that may express immunoreactive maternal proteins not shared by the child. Loss of tolerance to non-shared maternal antigens could lead to chronic activation of the child’s T lymphocytes, with subsequent autoimmunity through a mechanism of epitope spreading, similar to the graft-versus-host disease seen after transplantation of parental stem cells in animal models.

## Methods

PBMC from 26 pediatric SLE patients and 35 age-matched controls were stimulated with maternal or unrelated donor PBMC for 5 days. Cytokine expression and proliferation in T cell subsets was assayed by flow cytometry. Maternal microchimerism was assayed by Real-Time QPCR amplification of non-shared maternal alleles using >100,000 genome equivalents of genomic DNA isolated from PBMC. SLE disease activity was assessed by SELENA-SLEDAI. Pregnancy history was obtained from 78 SLE patients, 56 healthy controls, 37 scleroderma, and 16 Raynaud Phenomenon patients by parental report.

## Results

T lymphocytes in patients with SLE were hyperactive in response to maternal cells. Proliferation of CD4^+^ T lymphocytes in response to maternal cells was increased 2-fold in SLE patients compared to controls, and IFN-γ production by CD4^+^ lymphocytes was increased in patients with active SLE (SLEDAI>4), but not in those in remission or controls. TNF-α production by CD4^+^ T lymphocytes specific for maternal cells correlated with SLE disease activity. Levels of maternal microchimerism were not increased in SLE patients with active disease, but rather tended to be lower (75.6 per million maternal cells in controls, SD 282, versus 3.3 in SLE, SD 11, P=0.06). Of controls tested, MMc was detected in 34%, compared to 23% of SLE patients (P=0.4). Of the seven SLE patients with MMc, three had a history of premature birth (<35 weeks) with low birth weight (LBW, range 1.96-2.27 Kg). Overall, SLE patients had an increased prevalence of prematurity (15.4%) and LBW (<2.5 Kg, 14.7%) compared to local controls (no prematurity or LBW was reported), patients with scleroderma (10.8% prematurity, 8.3% LBW), Raynaud Phenomenon without underlying systemic disease (6.3% prematurity, 6.3% LBW), or historic national frequencies (5-8%).

## Conclusion

Elevated CD4^+^ T lymphocyte responses to maternal cells are consistent with a model of persistent T lymphocyte activation by chimeric maternal cells within target organs. Low levels of MMc in the blood suggest immune-mediated elimination of chimeric maternal cells in the periphery. Increased maternal-fetal cell traffic during delivery of premature infants and/or development of tolerance to maternal antigens during the last trimester may influence later responses to engrafted maternal cells in the child.

## Disclosure

Anne M. Stevens: None; Matthew Crabtree: None; Gretchen Henstorf: None; J. Lee Nelson: None.

